# Epidemiology and genotype 3 subtype dynamics of hepatitis E virus in Belgium, 2010 to 2017

**DOI:** 10.2807/1560-7917.ES.2019.24.10.1800141

**Published:** 2019-03-07

**Authors:** Vanessa Suin, Sofieke E Klamer, Veronik Hutse, Magali Wautier, Marjorie Jacques, Mona Abady, Sophie Lamoral, Vera Verburgh, Isabelle Thomas, Bernard Brochier, Lorenzo Subissi, Steven Van Gucht

**Affiliations:** 1 National Reference Centre of Hepatitis Viruses, Viral Diseases, Infectious Diseases in Humans, Sciensano, Brussels, Belgium; 2 Epidemiology of Infectious Diseases, Epidemiology and Public Health, Sciensano, Brussels, Belgium; 3 European Program for Intervention Epidemiology Training, European Centre for Disease Prevention and Control, Stockholm, Sweden; 4 European Program for Public Health Microbiology Training, European Centre for Disease Prevention and Control, Stockholm, Sweden

**Keywords:** hepatitis E virus, zoonoses, surveillance, epidemiology, food-borne infections, Belgium, epidemiology, hepatitis E, laboratory, laboratory surveillance, viral infections, zoonotic infections

## Abstract

**Background:**

Hepatitis E virus (HEV) is an emerging public health concern in high-income countries and can cause acute and chronic hepatitis. Reported numbers of indigenously acquired HEV infection have increased in the past decade in many European countries. Since 2010, the National Reference Centre (NRC) for Hepatitis Viruses has been testing samples of suspected hepatitis E cases in Belgium.

**Aim:**

In this surveillance report, we present the epidemiological trends of symptomatic HEV infections in Belgium, from the distribution by age, sex and geography to the molecular characterisation of the viral strains.

**Method:**

Serum samples of suspected cases sent to the NRC between 2010 and 2017 were analysed for the presence of HEV-specific IgM and RNA. Virus was sequenced for genotyping and phylogenetic analysis in all samples containing sufficient viral RNA.

**Results:**

The NRC reported an increase in the number of samples from suspected cases (from 309 to 2,663 per year) and in the number of laboratory-confirmed hepatitis E cases (from 25 to 117 per year). Among 217 sequenced samples, 92.6% were genotype 3 (HEV-3), followed by 6.5% of genotype 1 and 0.9% of genotype 4. HEV-3 subtype viruses were mainly 3f, 3c and 3e. HEV-3f was the most common subtype until 2015, while HEV-3c became the most common subtype in 2016 and 2017.

**Conclusion:**

The increasing trend of HEV diagnoses in Belgium may be largely explained by increased awareness and testing.

## Background

Hepatitis E virus (HEV) infection is a worldwide cause of viral hepatitis. From its discovery in 1983 until recently, HEV was mainly considered as a waterborne disease endemic in low- and middle-income countries [1]. In the past decade, infections with indigenously acquired HEV have increasingly been reported in Europe, and the virus is thought to be transmitted, among other things, through undercooked deer, pork meat and shellfish [2]. Infections in humans are caused by genotypes 1 (HEV-1) and 2 (HEV-2), obligate human pathogens, and genotypes 3 (HEV-3), 4 (HEV-4) and 7 (HEV-7), mostly zoonotic. HEV-1 and HEV-2 detected in patients living in Europe are mostly travel-acquired infections, whereas HEV-4 is found mostly in South East Asia and China and HEV-3 is endemic worldwide [2].

Infections with HEV-3 and HEV-4 are often asymptomatic and otherwise typically cause mild to moderate disease. Rarely, the virus can provoke fulminant hepatitis in acute cases, and patients with underlying chronic liver disease are mainly at risk [3]. HEV can also cause severe liver damage in chronically infected patients and untreated patients with underlying diseases [3].

The seroprevalence in blood donors in countries of the European Union (EU) ranges from 2% to 50% [4-8]. The seroprevalence in some regions of Belgium was estimated around 15% [9]. Several studies in Europe have found up to 90% HEV-seropositive pigs on farms and HEV RNA was detected in up to 45% of pigs at slaughterhouses [10-13].

The epidemiology of HEV infections among the Belgian population remains poorly described. In 2010, the National Reference Centre (NRC) for Hepatitis Viruses was created at Sciensano by the national health authorities. The NRC is responsible for laboratory confirmation of suspected hepatitis E cases and genotyping of the causative virus in clinical samples. The aim of this study was to provide an overview of the epidemiological situation of HEV in Belgium from 2010 until 2017.

## Methods

### Patient samples

Suspected cases were defined as any person whose sample was sent to the NRC for HEV testing. Confirmed cases were defined as persons with an IgM- and/or RT-qPCR-positive sample.

This study included all laboratory tests performed on clinical samples of suspected cases that were submitted to the NRC for HEV diagnosis between 1 January 2010 and 31 December 2017. Serum samples originated from hospitals and peripheral laboratories throughout Belgium and were submitted by general practitioners or internal medicine specialists. Belgian clinicians can send a clinical sample to the NRC, either to obtain a primary diagnosis or to obtain a confirmation diagnosis for HEV serology and/or HEV RNA. Similarly, primary laboratories are asked to send all positive and equivocal samples to the NRC for confirmation of the diagnosis, genotyping and epidemiological purpose. Primary laboratories may also send suspected samples to obtain a primary diagnosis when HEV serology and/or HEV RNA testing is not routinely performed in their laboratory. The submission of samples to the NRC is voluntary. The costs of the confirmation diagnosis and genotyping are covered by the federal NRC programme. We quantified the proportion of clinical laboratories that send samples for HEV testing to the NRC. Overall, 84% of the laboratories (86% of hospital laboratories and 83% of primary care laboratories) participated at least once during the study period.

We assumed that all samples received by the NRC were from patients who presented with clinical signs of viral hepatitis and/or increased biochemical liver values, or from patients with increased risk at developing disease and/or complications when infected (e.g. liver transplant patients). The following data were collected for each sample on the request form: requested analysis (IgG, IgM, qPCR or sequencing), date of birth, sex, postal code, hospitalisation status, travel history, clinical data, sampling date and referring laboratory and/or clinician. HEV serology and RT-qPCR were performed upon request and when sufficient sample volume was present. RT-qPCR was performed routinely on all IgM-positive samples. Cases were defined as laboratory-confirmed if the serum submitted to the NRC for HEV diagnostics was positive for HEV IgM and/or RNA.

### Hepatitis E virus serology

HEV serology (IgM) was performed using the commercial enzyme immunoassay RecomWell HEV IgG/M (Mikrogen Diagnostik, Germany; specificity: 98.6%, sensitivity: 98.9%) from 2010 to 2015 and the Wantai IgM assay (Sanbio, Japan; specificity: 95.3–100%, sensitivity: 97.1%) from 2016 to 2017. Signal to cut-off ratios were interpreted as prescribed. All positive and equivocal results obtained with the Mikrogen ELISA were considered positive only when confirmed by Western blot analysis (RecomBlot HEV IgG/M; Mikrogen Diagnostik, Germany). All equivocal results obtained with the Wantai IgM ELISA were considered positive only when the samples tested positive a second time.

Positive and negative controls from the kits and an external positive quality control were included in each assay. The external positive control for HEV IgM consisted of a pool of several positive anti-HEV IgM sera collected in the past. All positive HEV IgM were also analysed by qPCR.

### Detection and sequencing of hepatitis E virus RNA

HEV RNA was extracted from serum samples using the QIAamp Viral RNA mini kit (Qiagen, Germany). Specific HEV RNA was analysed using the commercial RealStar HEV qRT-PCR Kit 1.0 (Altona Diagnostics, Germany). For each qRT-PCR assay, several controls were analysed in duplicate: a positive control from the Real Star kit, an external positive and negative control and an extraction control to identify possible qRT-PCR inhibition and to confirm the integrity of the reagents of the kit. The external positive control used was the World Health Organization standard for HEV RNA nucleic acid amplification 10 × diluted (National Institute for Biological Standards and Control, England). The external negative control used was RNase-free water sample. Results were interpreted according to the manufacturer’s protocol. The extraction control was RNase P detection with primers from Hummel et al. [14].

In case of positive qRT-PCR (from 2010 to 2016), HEV was genotyped according to a protocol adapted from Huang et al. [15]. Since HEV strains are genetically heterogenic, a universal HEV nested RT-PCR assay with degenerated HEV primers was developed to detect genetically divergent strains of HEV. Two sets of degenerated HEV primers were used for the universal nested RT-PCR assay: external primer set 3156N (forward, 5’-AATTATGCC(T)CAGAC(T)CGG(A)GTG-3’) and 3157N (reverse, 5’-CCCTTA(G)TCC(T)TGCTGA(C)GCATTCTC-3’) and internal primer set 3158N (forward, 5’-GT(A)ATGCTT(C)TGCATA(T)CATGGCT-3’) and 3159N (reverse, 5’-AGCCGACGAAATCAA TTCTGC-3’). The expected product of the universal nested RT-PCR consisted of 348 bp in the ORF2 region of the HEV genome [15]. From 2017 onwards, a more sensitive genotyping protocol was used as described by Boxman et al. [16].

Positive amplicons were sequenced using the big dye terminator v.3.1 cycle sequencing kit (Applied Biosystems, United States (US)) and analysed on an ABI Prism 3130 Genetic Analyzer (Applied Biosystems, US). Sequences of PCR products were determined for both DNA strands and were uploaded to HEVnet (https://www.rivm.nl/en/hevnet), an EU database for HEV sequences hosted by the Dutch National Institute for Public Health and the Environment.

### Data analysis

The number of suspected and confirmed cases per year and month was based on the receipt date of the sample at the NRC. Multiple samples from the same patient in the same year were reduced to a single record. The confirmation ratio was calculated as the confirmed cases divided by the suspected cases within the same period. Trends over time of suspected and confirmed cases were modelled with linear regression analysis. The slope coefficients of the standardised data for suspected and confirmed cases were compared in linear regression analysis. Wilson 95% confidence intervals (CI) were calculated for proportions and Pearson’s chi-squared test was used to compare proportions. STATA 14.0 and SAS were used for the statistical analyses.

### Phylogenetic analysis

Phylogenetic trees included the 348 bp sequences of the ORF2 genes characterised at the NRC in patient’s clinical samples, the HEV reference strains from Smith et al. [17] and selected animal and human HEV sequences available in GenBank. Sequence comparison, alignments and phylogenetic trees were realised using CLC main workbench 7.8.1 and MEGA 7 software [18]. Sequences differing at more than 1% of nucleotide positions were analysed using the maximum likelihood method based on the Tamura-Nei model using MEGA 7 software. The confidence values of the internal nodes were calculated by performing 1,000 bootstrap analyses. When the sequences most closely related to Belgian isolates were reference strains, an indication of nucleotide p-distance was reported.

### Ethical statement

The study is covered by the official mandate of the National Reference Centre for Hepatitis Viruses to collect and analyse epidemiological and clinical data of hepatitis E cases in Belgium as part of the national surveillance plan and promotion of public health. Considering the retrospective and non-interventional nature of the study, approval of an ethics commission or individual informed consent were not required, in agreement with the Belgian Law from 7 May 2004 concerning experiments on people.

## Results

### Characteristics of hepatitis E patients

In 2010, 25 of 309 tested samples (8.1%) were from laboratory-confirmed hepatitis E cases, whereas in 2017, 117 of 2,663 tested samples (4.4%) were from laboratory-confirmed cases (Table 1). The overall confirmation ratio was stable between 2012 and 2017, ranging between 3.5% and 4.8%. Overall, 37% of the 417 confirmed cases were only IgM-positive (and PCR-negative) while the remaining 63% of cases were PCR-positive (and IgM-positive or -negative; Table 1). Hepatitis E was mainly detected in 40–64 year-old adults and rarely in children and adolescents. Men were more affected than women in this age group (Table 1). Overall, the annual sex ratio (male/female) varied between 1.6 and 4.0 during the study period. When looking at the three geographical regions of Belgium (Flanders, Wallonia and Brussels), inhabitants of Flanders represented 48% (4,172/8,651) of the suspected cases and 47% (193/408) of confirmed cases, while inhabitants of Wallonia represented 31% (2,688/8,651) of suspected samples and 43% (175/408) of confirmed cases. Finally, inhabitants of Brussels represented 21% (1,791/8,651) of the suspected cases and 10% (40/408) of confirmed cases (Table 1). Relative to the population, Wallonia was slightly oversampled compared with Flanders, whereas the number of tests relative to the population size was almost twice as high in Brussels (data not shown).

**Table 1:** Number of hepatitis E suspected cases, laboratory-confirmed cases (IgM+/PCR− and PCR+) and successfully sequenced cases per year, age group, sex and region of residence, Belgium, 2010–2017 (n = 8,941)

	Year	Suspected cases	Column percentages of suspected cases^a^	Confirmed cases (IgM+/PCR−)	Confirmed cases (PCR+ )	Sequenced PCR+ cases	Total confirmed cases	Column percentages of confirmed cases^a^
Year of receipt at National Reference Centre	2010	309	3.5	18	7	7	25	6.0
	2011	466	5.2	16	17	17	33	7.9
	2012	579	6.5	13	15	14	28	6.7
	2013	687	7.7	13	19	15	32	7.7
	2014	1,039	11.6	9	27	26	36	8.6
	2015	1,384	15.5	24	40	24	64	15.3
	2016	1,814	20.3	25	57	45	82	19.7
	2017	2,663	29.8	36	81	69	117	28.1
	**Total**	**8,941**	**100.0**	**154**	**263**	**217**	**417**	**100.0**
Age group	< 20	421	5.9	3	0	0	3	0.7
	20–39	1,813	25.6	35	36	32	71	17.2
	40–64	3,403	48.0	87	163	133	250	60.5
	≥ 65	1,448	20.4	28	61	49	89	21.5
	Missing	1,856	NA	1	3	3	4	NA
Sex	Male	3,915	51.9	96	183	153	279	67.4
	Female	3,634	48.1	56	79	62	135	32.6
	Missing	1,392	NA	2	1	2	3	NA
Region of residence	Flanders	4,172	48.2	75	118	98	193	47.3
	Wallonia	2,688	31.1	63	112	90	175	42.9
	Brussels	1,791	20.7	12	28	27	40	9
	Missing	290	NA	4	5	2	9	NA

IgM+: IgM-positive; NA: not applicable; PCR+: PCR-positive; PCR−: PCR-negative.

^a^ Column percentages were calculated excluding the records with missing values for the analysed characteristic.

**Figure 1:**
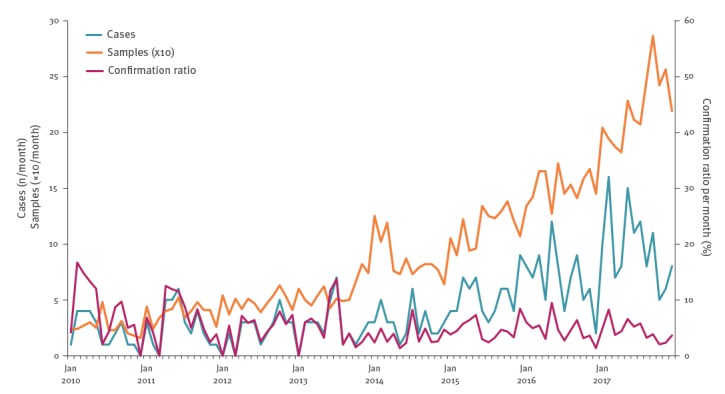
Number of hepatitis E suspected samples, laboratory-confirmed cases and confirmation ratio per month, Belgium, 2010–2017 (n = 8,941)

The number of suspected cases, laboratory-confirmed cases and the confirmation ratio per month are shown in Figure 1. The number of suspected and confirmed cases increased significantly over time between 2010 and 2017 in the linear regression (both p values < 0.001). The slope coefficients of both standardised trends were not significantly different.

### Genotyping and phylogenetic analysis

Genotyping was successful for 217 of 263 PCR-positive samples (82.5%), which represented 52.0% of all laboratory-confirmed cases (217/417; Table 1). HEV-3 was the most common genotype (92.6%, n = 201), followed by HEV-1 (6.5%, n = 14) and HEV-4 (0.9%, n = 2; Table 2). For three confirmed cases, a recent travel history outside Europe was reported and they were all infected with HEV-1. Among the 201 HEV-3 sequences, subtypes 3f (50.7%, n = 102) and 3c (39.8%, n = 80) were found most often, followed by 3e (6.4%, n = 13), whereas 3a and 3h with 1.0% (n = 2) and 1.5 % (n = 3), respectively, were rarely detected (Table 3). During the period from 2010 to 2015, 3f accounted for 58.7% of all genotype 3 sequences, while 3c accounted for 27.2% and other HEV-3 subtypes for 14.1%. These proportions changed during the period from 2016 and 2017: 3c increased to account for 50.5%, 3f decreased to 44.0% and other HEV-3 subtypes decreased to 5.5% (chi-squared p value < 0.001, Table 3).

**Table 2:** Hepatitis E virus infections by genotype, Belgium, 2010–2017 (n = 217)

Genotype	2010	2011	2012	2013	2014	2015	2016	2017	Total
HEV-1	2	3	2	0	0	2	4	1	14
HEV-3	5	14	10	15	26	22	41	68	201
HEV-4	0	0	2	0	0	0	0	0	2

**Table 3:** Hepatitis E virus genotype 3 infections by subtype, Belgium, 2010–2017 (n = 201)

Genotype	2010	2011	2012	2013	2014	2015	2016	2017	Total
HEV-3a	0	0	0	0	0	2	0	0	2
HEV-3c	1	2	0	4	9	9	20	35	80
HEV-3e	1	2	1	3	2	0	1	3	13
HEV-3f	3	10	9	8	13	11	19	29	102
HEV-3h	0	0	0	0	1	0	1	0	2
HEV-3i	0	0	0	0	0	0	0	1	1
HEV-3ra	0	0	0	0	1	0	0	0	1

**Figure 2:**
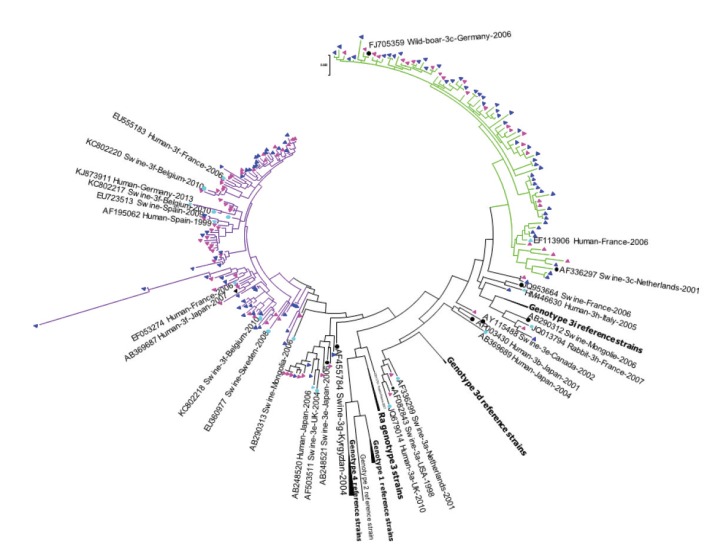
Phylogenetic analysis of hepatitis E virus genotype 3 isolates, Belgium, 2010–2017 (n = 186 human sequences)

HEV: hepatitis E virus.

Maximum-likelihood phylogenetic tree of a 348 bp fragment from open reading frame 2. Genetic distances were calculated using the Tamura Nei model. GenBank accession numbers are shown for each HEV reference strain used in the phylogenetic analysis and are noted as follows: Accession number_Host-HEV subtype-Country-Year. Triangles represent human HEV sequences and circles animal sequences.

Green lines: all HEV-3c isolates; purple lines: all HEV-3f isolates. Rose symbols: HEV sequences from 2010 to 2015; dark blue symbols: HEV sequences from 2016 to 2017; black symbols: HEV reference strains according to Smith et al. [17]; light blue symbols: other human and animal HEV sequences.

**Figure 3:**
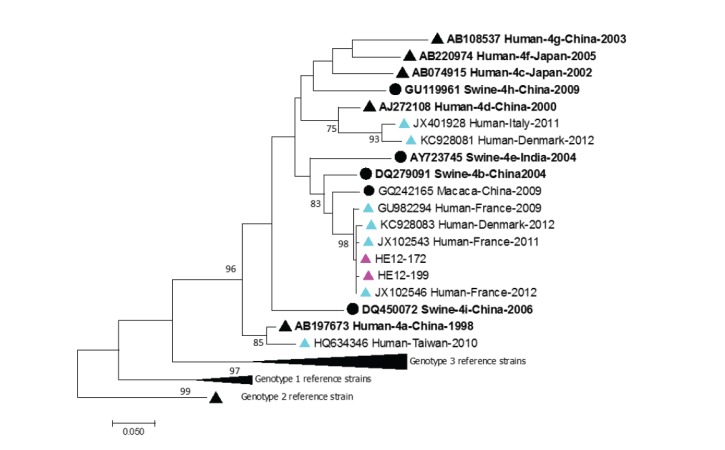
Phylogenetic analysis of hepatitis E virus genotype 4 isolates, Belgium, 2010–2017 (n = 14 human sequences)

Some Belgian HEV-3c strains clustered with previously described European or American human or swine HEV isolates [19]. Belgian HEV-3f isolates were closely related to previously described swine or human strains from European countries and Japan (2007). Both Belgian HEV-4 isolates from 2012 were most closely related to human HEV-4 strains from France isolated in 2011 and 2012 (Figure 3).

HEV: hepatitis E virus.

Maximum-likelihood phylogenetic tree of a 348 bp fragment from open reading frame 2. Genetic distances were calculated using the Tamura Nei model. Only bootstrap values > 70 are reported. GenBank accession numbers are shown for each HEV reference strain used in the phylogenetic analysis and are noted as follows: Accession number_Host-HEV subtype-Country-Year. Triangles represent human HEV sequences and circles animal sequences.

Rose symbols: 2012 Belgian HEV-4 sequences; black symbols: HEV reference strains according to Smith et al. [17]; light blue symbols: other human and animal HEV sequences.

### Geographical distribution of hepatitis E virus-confirmed cases

For further analysis, known HEV-1 and HEV-4 cases were excluded from the confirmed cases. The resulting nation-wide HEV-3 confirmation ratio was highest in 2010 (7.5%), lowest in 2014 (3.4%) and ranged between 4.0 and 4.5% for the period from 2015 to 2017. A significant difference in the confirmation ratio was only detected between the years 2010 (95% CI: 4.8–11.0) and 2014 (95% CI: 2.4–4.7; Table 4).

**Table 4:** Number of tested samples and confirmed cases, excluding all known HEV-1 and HEV-4 cases, in the regions Flanders, Wallonia and Brussels per year, Belgium, 2010–2017 (n = 8,923)

Region of residence	Year	Number of tested samples^a^	Confirmed cases (excluding HEV-1 and HEV-4)^b^	Proportion confirmed (%)	95% confidence interval
Flanders	2010	135	11	8.1	4.6–14.0
	2011	214	18	8.4	5.4–12.9
	2012	254	10	3.9	2.2–7.1
	2013	315	15	4.8	2.9–7.7
	2014	443	17	3.8	2.4–6.1
	2015	671	30	4.5	3.2–6.3
	2016	957	32	3.3	2.4–4.7
	2017	1,176	53	4.5	3.2–6.3
Wallonia	2010	122	10	8.2	4.5–14.4
	2011	147	12	8.2	4.7–13.7
	2012	201	14	7.0	4.2–11.4
	2013	196	16	8.2	5.1–12.8
	2014	301	15	5.0	3.0–8.1
	2015	402	27	6.7	4.7–9.6
	2016	511	34	6.7	4.8–9.2
	2017	805	44	5.5	4.1–7.3
Brussels	2010	45	2	4.4	1.2–14.8
	2011	91	0	0.0	0.0–4.1
	2012	97	0	0.0	0.0–3.8
	2013	155	1	0.6	0.1–3.6
	2014	278	3	1.1	0.4–3.1
	2015	280	5	1.8	0.8–4.1
	2016	276	7	2.5	1.2–5.1
	2017	561	14	2.5	1.5–4.1
Overall^c^	2010	307	23	7.5	4.8–11.0
	2011	463	30	6.5	4.4–9.1
	2012	575	24	4.2	2.7–6.2
	2013	687	32	4.7	3.2–6.5
	2014	1,039	35	3.4 ^d^	2.4–4.7
	2015	1,382	62	4.5	3.5–5.7
	2016	1,809	73	4.0 ^d^	3.2–5.1
	2017	2,661	111	4.2 ^d^	3.4–5.0

^a^ Excluding tested samples identified as HEV-1 and HEV-4.

^b^ The confirmed cases that were not sequenced are included.

^c^ Including 290 suspected cases with unknown region of residence spread over all years.

^d^ Including nine confirmed cases with unknown region of residence spread over the years 2014, 2016 and 2017.

During the study period, the overall confirmation ratio was lowest in Brussels (1.8, 95% CI 1.3–2.5) and highest in Wallonia (6.4; 95% CI: 5.5–7.4); in Flanders, it was 4.5 (95% CI: 3.9–5.1). We observed for each year a higher confirmation ratio in Wallonia (range: 5.0–8.2%) compared with Flanders (range: 3.3–4.8%) and this difference was significant for the year 2016 (3.3%; 95% CI: 2.4–4.7 in Flanders vs 6.7%; 95% CI: 4.8–9.2 in Wallonia; Table 3). The confirmation ratios for Wallonia were also significantly higher than those for Brussels for the years 2011 to 2013 and 2015.

## Discussion

The epidemiology of symptomatic HEV infection among people in Belgium is poorly described. Here, we provide an overview of the epidemiological situation of HEV in laboratory-confirmed patients and an analysis of strains circulating in Belgium between 2010 and 2017. We report an increase in the number of hepatitis E cases diagnosed at the NRC and a simultaneous increase in the number of tested samples. Based on our data, increased awareness and testing are the most likely contributors to the rise in the number of confirmed hepatitis E cases in Belgium. The number of reported confirmed hepatitis E cases consistently and simultaneously increased in many European countries during the last decade [20,21]. However, a recent study from Germany showed a slight decline in seroprevalence between 1998 and 2010, suggesting a decrease in the seroincidence during this period [22]. As expected, we found that men represented two thirds of the confirmed cases and the median age was more than 50 years. In Belgium, it is unclear why the highest number of cases relative to the number of tested samples were recorded in the Walloon region. Interestingly, a nationwide survey of HEV infections among blood donors in France found the north-east region close to the border with the Walloon region as one of the three areas of France with highest HEV seroprevalence [23]. In Brussels, the number of tests relative to the population size was almost double compared with the two other regions (where they were roughly comparable, data not shown). This might be explained by higher awareness among physicians or possibly the different population characteristics (e.g. higher incidence of viral hepatitis of the types A, B and C).

Phylogenetic analysis identified HEV-3 as the main circulating genotype in Belgium, while HEV-1 and HEV-4 were detected sporadically. For most cases with HEV-3 infection, information about travel history was missing and it was thus not possible to exclude that the infection was acquired abroad. However, our molecular data suggest that the HEV-3 infections were acquired in Europe.

In England and Wales, the Netherlands and Germany, the most commonly identified HEV-3 subtype is 3c, while the main one in Spain and France is 3f [20]. In Belgium, 3f was the most commonly identified subtype until 2015, whereas 3c was the most commonly identified subtype in 2016 and 2017. This trend was also observed in England and Wales between 2010 and 2012, where the number of subtype 3c strains identified from patients exceeded that of subtypes 3e, 3f and 3g [24]. HEV-3 strains from cases in Belgium tend to be closely related to the strains present in European pigs and wildlife (wild boar and deer). Products such as raw pork liver, raw pork sausages and pork pâté have been identified previously as likely sources of transmission [25-27]. Interestingly, all swine HEV sequences obtained by Thiry et al. in 2014 from Belgian swine sera belonged to HEV-3f, similar to the dominant subtype found in human cases until 2015 in this study [10].

One of the limitations of this study is that we used IgM data from two different ELISA assays with different specificity and sensitivity. The fact that the proportion of IgM-positive to PCR-negative cases did not increase after the introduction of the Wantai assay makes us confident that this switch has not considerably affected our diagnoses over the years. An additional limitation of this study is that the submission of samples to the NRC is voluntary, but over the years, 84% of all Belgian diagnostic centres participated to this surveillance system at least once. Furthermore, we had limited clinical and epidemiological data available from our cases (e.g. travel history and underlying comorbidities). The asymptomatic or mild presentation of most HEV infections implies that laboratory-confirmed cases are not representative of all HEV infections. Unfortunately, recent nation-wide HEV seroprevalence studies are lacking, which would have completed the epidemiological observations.

## Conclusion

We observed an increasing trend of HEV-confirmed cases over the study period, and a simultaneous increase in the number of suspected samples, with a tendency of higher confirmation ratios observed for the south of Belgium (Wallonia) compared with the other regions. We think that this increasing trend is mainly due to increased awareness and testing because of the simultaneous increase in the number of suspected samples. We cannot exclude minor dynamics in disease burden over the past years, but a pronounced recent increase in the overall disease burden may be excluded because the confirmation ratio has been stable since 2012. The regional differences in confirmation ratios may guide further investigations about exposures (e.g. food habits) and risk factors (e.g. liver disease).

The majority of the cases were typed as HEV-3, of the subtypes 3f and 3c. The changing epidemiology of HEV-3c may warrant further investigation, e.g. to study the presence of this subtype among livestock and wild boars. More studies on the epidemiology and subtypes of HEV in animal reservoirs and in food may identify transmission routes in Belgium. Consumption of products containing raw or undercooked pig liver, blood and meat products may represent the major risk factors for human HEV infections, and adaptations of food production processes (e.g. heat treatment) are highly recommended in order to improve food safety.
